# Transplantation rénale au Maroc: l'hémodialysé et son entourage sont-ils suffisamment informés?

**DOI:** 10.11604/pamj.2014.19.365.5257

**Published:** 2014-12-09

**Authors:** Béfa Noto-Kadou-Kaza, Kossi Akomola Sabi, Ghislain Imangue, Mays Hadi Al-Torayhi, Eyram Yoan Makafui Amekoudi, Claude Mawufemo Tsevi, Mohamed Zamd, Ghislaine Medkouri, Mohamed Gharbi Benghanem, Benyounes Ramdani

**Affiliations:** 1 Service de Néphrologie, de Dialyse et de Transplantation Rénale du CHU Ibn Rochd de Casablanca-Maroc; 2 Service de Néphrologie et d'Hémodialyse du CHU Sylvanus Olympio de Lomé-Togo

**Keywords:** Transplantation rénale, niveau de connaissance, hémodialysés, entourage, kidney transplant, knowledge, he;modialysis, caretakers

## Abstract

**Introduction:**

La transplantation rénale constitue le traitement idéal de l'insuffisance rénale chronique. Cependant il existe une insuffisance de donneurs contrairement aux receveurs dont le nombre ne cesse de s'accroitre. La méconnaissance de la transplantation par les hémodialysés et leur entourage pourrait être l'une des causes. Notre but était d’évaluer les connaissances et opinions de l'hémodialysé et de son entourage sur la transplantation rénale.

**Méthodes:**

L'enquête menée en Aout 2013 avait inclus 83 hémodialysés de notre centre et 70 membres de leur entourage. Ils ont été soumis à un questionnaire qui portait sur les thèmes suivants: statut socio-économique, volonté d’être transplanté ou d’être donneur, avantages de la transplantation rénale, point de vue de la religion sur la transplantation. Aucun des individus interrogés n'avait jamais fait l'objet d'une transplantation ou d'un don d'organe.

**Résultats:**

Parmi 83 hémodialysés on notait 49,4% de femmes avec une moyenne d’âge de 41,4±12 ans. Le niveau socio-économique bas représentait 66,7%. Le manque d'information chez était estimé à 62,7%. Seuls 41% se disaient être candidat à la transplantation rénale mais 12% seulement était inscrit sur la liste d'attente de greffe. La transplantation était estimée plus couteuse que l'hémodialyse par 50,6% des patients et seuls 71,1% estimaient qu'elle offrait une meilleure vie. Pour 20,5%, l'Islam s'opposerait au don cadavérique et pour 10,9% au don vivant. Parmi les 70 membres de l'entourage interrogé il y avait 56,8% de femmes; la moyenne d’âge était de 44,4±10,5 ans. Le niveau économique bas représentait 52,3%. Le manque d'information était estimé à 61,4%. Pour 56,8% la vie serait impossible avec un seul rein. Seuls 13,6% étaient inscrit sur le registre de don. Pour 45,5% l'Islam s'opposerait au don cadavérique.

**Conclusion:**

Il importe d'intensifier la sensibilisation des hémodialysées et leur famille sur la transplantation rénale.

## Introduction

La transplantation rénale (TR) constitue de nos jours le traitement idéal de l’insuffisance rénale chronique. En effet contrairement aux autres modalités thérapeutiques, elle permet non seulement de prolonger la vie, mais aussi assure une meilleure qualité de vie et une réduction à long terme du cout de prise en charge de ces patients [1–4]. Cependant dans tout le monde entier, il se pose le problème majeur de l’inadéquation entre le nombre de receveurs sans cesse croissant comparativement à celui des donneurs [5]. Au Maroc, malgré le peu d’hémodialysés qui postulent à la transplantation rénale, il se pose aussi des difficultés de disponibilité de donneurs vivants, et surtout une rareté de donneurs cadavériques. La méconnaissance de la part des patients et, de leur entourage des indications et contres indications médicales, de la législation et du point de vue de la religion sur la transplantation rénale pourrait expliquer en partie cette situation. Notre étude avait pour but d’évaluer le niveau de connaissances ainsi que l’opinion de l’hémodialysé et de son entourage sur la transplantation rénale.

## Méthodes

Il s’était agi d’une enquête monocentrique menée en Aout 2013 dans le centre d’hémodialyse du Centre Hospitalier Universitaire Ibn Rochd de Casablanca. Elle avait inclus les hémodialysés ainsi que les membres de leur entourage qui avaient donné librement leur accord de participation. N’ont pas été inclus ceux ayant refusé, ainsi que ceux ayant fait antérieurement soit objet d’une transplantation ou d’un don d’organe.

Ils ont été tous soumis à un questionnaire élaboré en langue française et traduite en langue arabe, validé par notre service. Ce questionnaire était à la fois à choix simples et à choix multiples ainsi que des questions à réponse ouverte courte. Les patients ont été directement interrogés par un investigateur soit avant, pendant ou après leur séance de dialyse. Un membre de l’entourage de chaque malade a été convoqué au centre spécialement pour l'enquête.

Les thèmes abordés dans le questionnaire portaient sur le statut socio-économique, les données d’hémodialyse (HD), la volonté d’être transplanté ou d’être donneur, la connaissance sur les avantages de la transplantation ainsi que sur les risques liés au don, la connaissance du point de vue de la religion sur la transplantation et sur le don vivant et le don cadavérique, l’opinion personnelle sur la transplantation et sur le don. L’évaluation du niveau socio-économique était basée sur les critères décris en 2009 par le Haut-Commissariat au Plan (HCP) [6]. Les données ont été saisies et analysées à l’aide du logiciel SPSS 18.0. Les variables quantitatives ont été exprimées en moyenne et en écart-type.

## Résultats

Sur un total de 103 hémodialysés que compte notre centre, 83 soit 80,60% avaient accepté participer à l’enquête. Dans l’entourage le taux de participation était de 70/83 membres convoqués soit 84,30%.

Parmi les 83 hémodialysés, on notait 49,4% de femmes. La moyenne d’âge était de 41,4±12 ans. Le niveau socio-économique bas était prédominant dans 66,70%. Seulement 7,2% avaient un niveau d’étude universitaire. La durée moyenne d’hémodialyse était de 12,30±6,5 ans (Tableau 1). Concernant la connaissance sur la transplantation rénale, 62,70% de ces hémodialysés avaient déclaré n’avoir aucune idée sur la transplantation rénale. Pour ceux qui étaient informés, seulement 48,39% disaient avoir reçu les informations de leurs néphrologues. Seuls 41% voudraient se faire transplanter dont 12% inscrit sur la liste d’attente de greffe. Près de 13,20% étaient absolument opposés à la transplantation rénale. Pour 50,60% des patients, la transplantation était plus couteuse que l’hémodialyse. Seulement 71,1% estimaient que la transplantation offrait offrait une meilleure qualité de vie comparée à l’hémodialyse. L’Islam s’opposerait au don cadavérique pour 20,50% des patients et au don vivant pour 10,90%. Seuls 12,40% étaient personnellement opposés au don cadavérique et 14,45% au don vivant (Tableau 2).

**Tableau 1:** Données épidémiologiques des hémodialysés

Données	Hémodialysés (n = 83)
**Caractéristiques des patients**	
Age (moyenne ± DS), année	41,4 ± 12
Sexe féminin, n (%)	41 (49,4)
**Niveau socio-économique, n(%)**	
Bas	55(66,3)
Moyen	27(32,5)
Elevé	1(1,2)
**Niveau d’étude, n(%)**	
Analphabète	25(30,12)
Primaire	28(33,7)
Secondaire	24(38,9)
Universitaire	6(7,2)
**Données d'hémodialyse**	
Durée dialyse (moyenne±DS), année	12,30 ± 6,5
Néphropathie initiale, n(%)	
Diabète	6(7,2)
Glomérulonéphrite chronique	23(27,72)
Néphropathie indéterminée	37(44,6)

**Tableau 2:** Connaissance et opinions des hémodialysés sur la transplantation rénale

Données	Hémodialysés (n = 83)
**Connaissance sur la TR, n(%)**	
Manque d'information	52(62,7)
**Origine de l'information**	
Néphrologues	15(48,39)
Médias (TV, Radio, etc)	10(32,26))
Infirmiers	6(19,35)
Autres patients	5(16,13)
TR plus couteuse que l'hémodialyse	42(50,6)
Qualité de vie du transplanté mieux que l'hémodialysé	59(71,1)
Volonté d’être transplanté	34(41)
Hésitation à la transplantation	38(45,8)
Opposition à la transplantation	11(13,2)
Point de vue de la religion sur la TR selon l'hémodialysé	17(20,5)
Islam opposé au don vivant	9(10,93)
Opposé au don cadavérique	10(12,4)
Opposé au don vivant	12(14,45)

TR : Transplantation Rénale

Parmi les 70 membres de l’entourage, il y avait une prédominance féminine avec 56,80%. La moyenne d’âge était de 44,4±10,5 ans. Le niveau socio-économique bas prédominait dans 52,30%. Seulement 18,60% avaient un niveau d’étude universitaire (Tableau 3). Le manque d’information sur la transplantation rénale était estimé à 61,40%. Les informations provenaient des néphrologues dans 48,15%. Pour 56,80% il serait impossible de vivre avec un seul rein. Seulement 35,70% s’étaient déclarés volontaire au don avec 13,60% inscrits sur le registre de don. Pour 45,50% l’Islam s’opposerait au don cadavérique et au don vivant pour 27,30%. De même 18,60% étaient personnellement opposés au don cadavérique et 22,80% au don vivant (Tableau 4).

**Tableau 3:** Données épidémiologiques de l'entourage

Données	Entourage (n = 70)
Age (moyenne±DS), année	44,4 ± 10,5
Sexe féminin, n (%)	40(56,8)
**Niveau socio-économique, n(%)**	
Bas	37(52,8)
Moyen	30(42,92)
Elevé	3(4,32)
**Niveau d’étude, n(%)**	
Analphabète	16(22,8)
Primaire	27(38,6)
Secondaire	14(20)
Universitaire	13(18,6)

**Tableau 4:** Connaissance et opinions de l'entourage sur la transplantation rénale

Données	Entourage (n = 70)
**Connaissance sur la TR, n(%)**	
Manque d'information	43(61,4)
**Origine de l'information**	
Néphrologues	13(48,15)
Médias (TV, Radio, etc)	12(44,44)
Infirmiers	4(14,81)
Autres patients	3(11,11)
Vie impossible avec un seul rein	40(57)
Volonté d’être donneur	25(35,7)
Inscription sur le registre de don	10(14,3)
**Point de vue de la religion sur la TR selon l'entourage**	
Islam opposé au don cadavérique	38(45,5)
Islam opposé au don vivant	19(27,3)
Opposé au don cadavérique	13(18,6)
Opposé au don vivant	

TR : Transplantation Rénale

## Discussion

Notre objectif était d’évaluer le niveau de connaissance de l’hémodialysé et de son entourage sur la transplantation rénale. De ce fait, cette étude présente quelques biais notamment lié à son caractère monocentrique. Aussi le faite que notre centre d’hémodialyse appartient à un service spécialisé dans la transplantation rénale laisserait penser que nos patients devraient être d’avantage informés sur la transplantation. Cependant nos résultats ont prouvés le contraire. De notre étude, se dégage un constat fondamental selon lequel: la méconnaissance de la transplantation rénale est une réalité chez nos hémodialysés et leur entourage comme d’ailleurs en témoignent les nombreuses preuves ci-après.

Près des deux tiers de nos hémodialysés (62,70%) et de leur entourage (61,40%) avaient déclarés n’avoir jamais reçu d’information concernant la transplantation rénale. Ce qui d’ailleurs semble être confirmé par Laouad et al. [7] dans une étude similaire mais multicentrique menée dans 3 centres d’hémodialyses du pays en 2011 qui avait rapporté un taux de méconnaissance de 52,50%. Par contre Kucirka et al. aux Etats Unis [8] ont rapporté en 2012 un taux nettement plus bas qui était de 30,1% parmi les insuffisants rénaux chroniques terminaux américains d’origine africaine.

Au Quatar en 2005, El-Shoubaki et al. [9] avaient eux aussi rapporté un taux d’ignorance de 30% mais dans la population générale. L’une des raisons qui expliqueraient un tel taux de méconnaissance élevé sur la TR au Maroc pourrait être l’insuffisance de travail d’information, d’éducation et de sensibilisation sur la transplantation rénale, de la part du personnel médical en général et en particulier des néphrologues au profit des hémodialysés et de leur famille. Aussi les énormes efforts de campagnes locales et nationales de masse sur la transplantation rénale consentit jusque là paraissent insuffisants.

Pour environ la moitié des patients la transplantation rénale couterait plus chère que l’hémodialyse. Et pourtant il est bien reconnu que la greffe rénale devient moins couteux que l’hémodialyse à partir de la deuxième année après la transplantation [10]. Plus de la moitié (57%) des membres de l’entourage avaient estimé qu’il serait impossible de vivre avec un seul rein alors qu’il est reconnu du point de vue physiologique qu’un rein peut à lui seul assurer l’épuration de tout l’organisme [11].

Près du quartz des hémodialysés et près de la moitié de l’entourage pensaient que l’Islam s’opposerait au don cadavérique. Laouad et Coll. [6] avaient rapporté un taux plus élevé de 65,9%. Pourtant dans tout le monde entier il n’existe aucune religion qui s’opposerait au don d’organe [12]. L’islam ne s’opposerait donc pas au don d’organe comme le stipule ce passage du Coran: « Quiconque donne la vie à une âme, c’est tout comme il avait donné la vie à toute l’humanité » [13].

D’ailleurs dans une étude menée auprès des religieux en Turquie en 2013 par Uskun et al. [14], 71,5% étaient favorables au don d’organes. Donc de tout ce qui précède, il apparait bien clair qu’il s’agit d’une mauvaise compréhension des textes religieux de la part des patients et de leur entourage qui leur font croire que l’Islam s’opposerait au don cadavérique. Aussi l’influence de la culture traditionnelle n’est serait pas négligeable. D’ailleurs en Asie, Randhawa avait estimé que la religion et la culture traditionnelle peuvent être des handicaps majeurs qui empêcheraient les familles au don d’organe [15].

## Conclusion

En dépit de tous les efforts déjà consentis, l'hémodialysé et son entourage au Maroc demeurent toujours peu informés sur la transplantation rénale. De même ils continuent par croire de façon erronée que l'Islam s'opposerait surtout au don cadavérique. Le renforcement de toutes les actions visant à informer, sensibiliser et à éduquer les populations en général et l'hémodialysé et son entourage en particulier, s'impose. L'IRC et sa famille doivent bénéficier dès le stade de début de la maladie d'une séance spéciale d'informations adéquates sur la transplantation rénale et surtout sur le don cadavérique qui a permis à bon nombre de pays d'augmenter la disponibilité des greffons.
